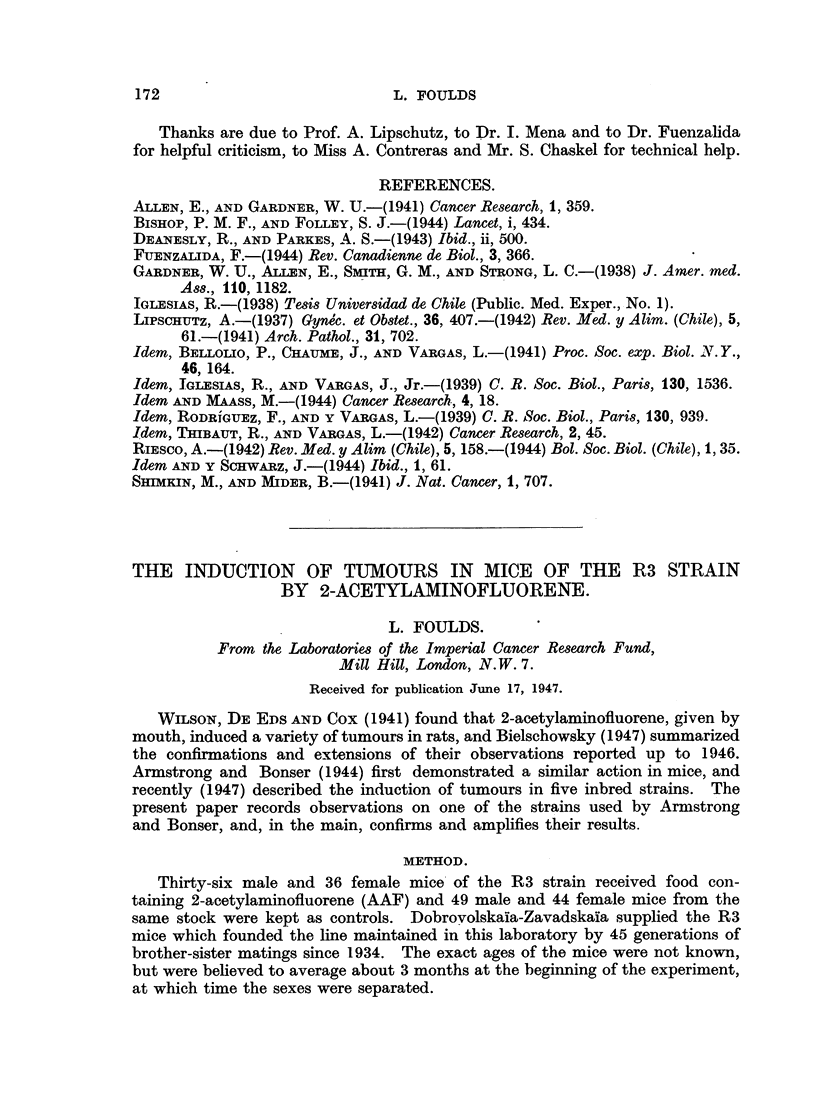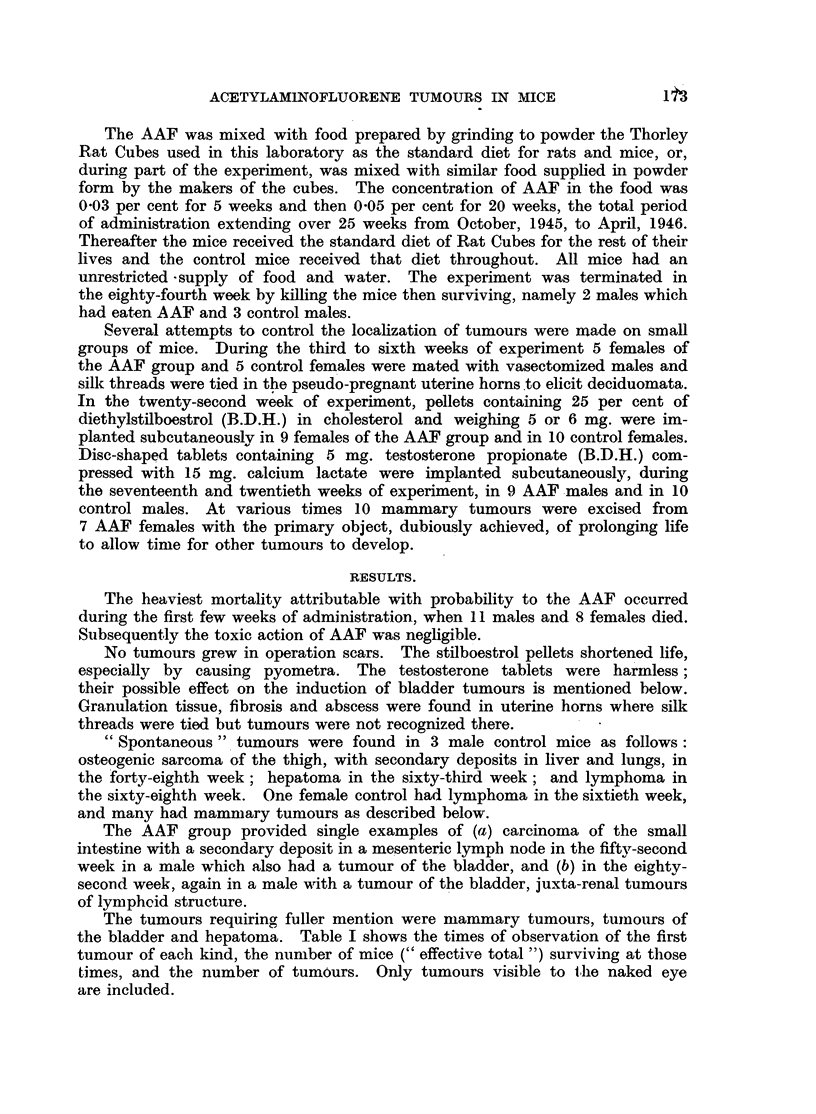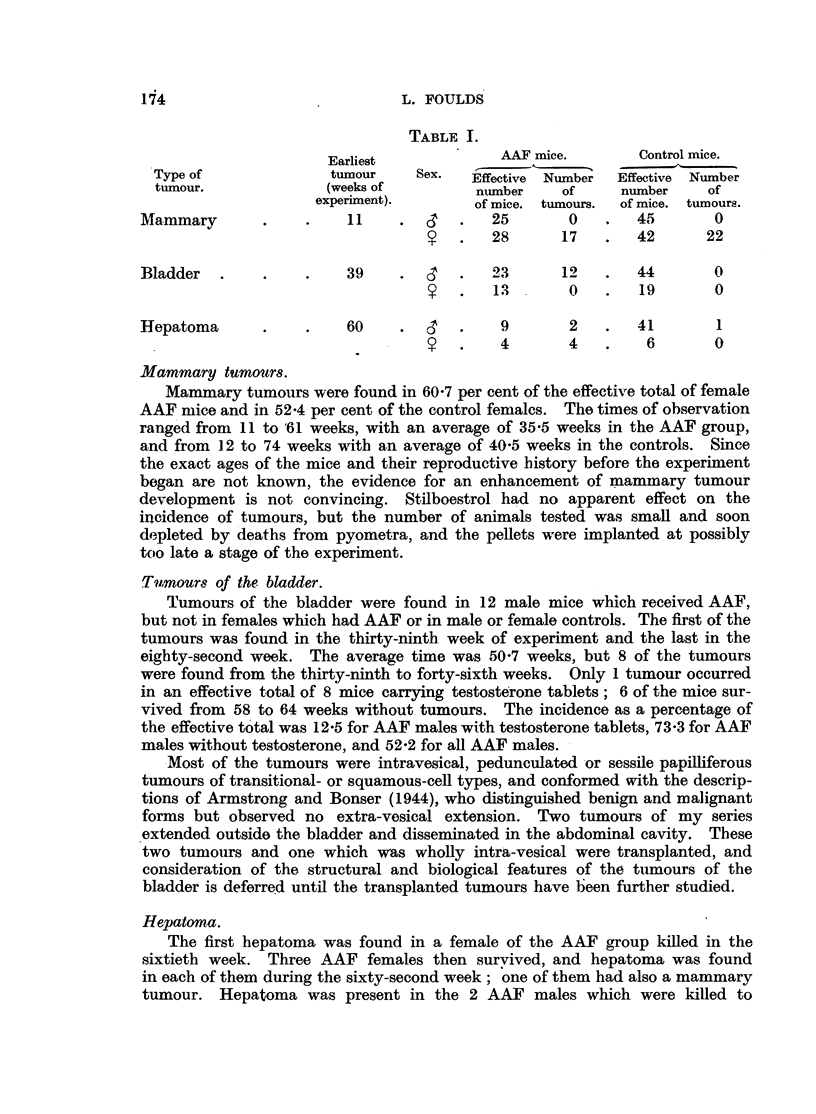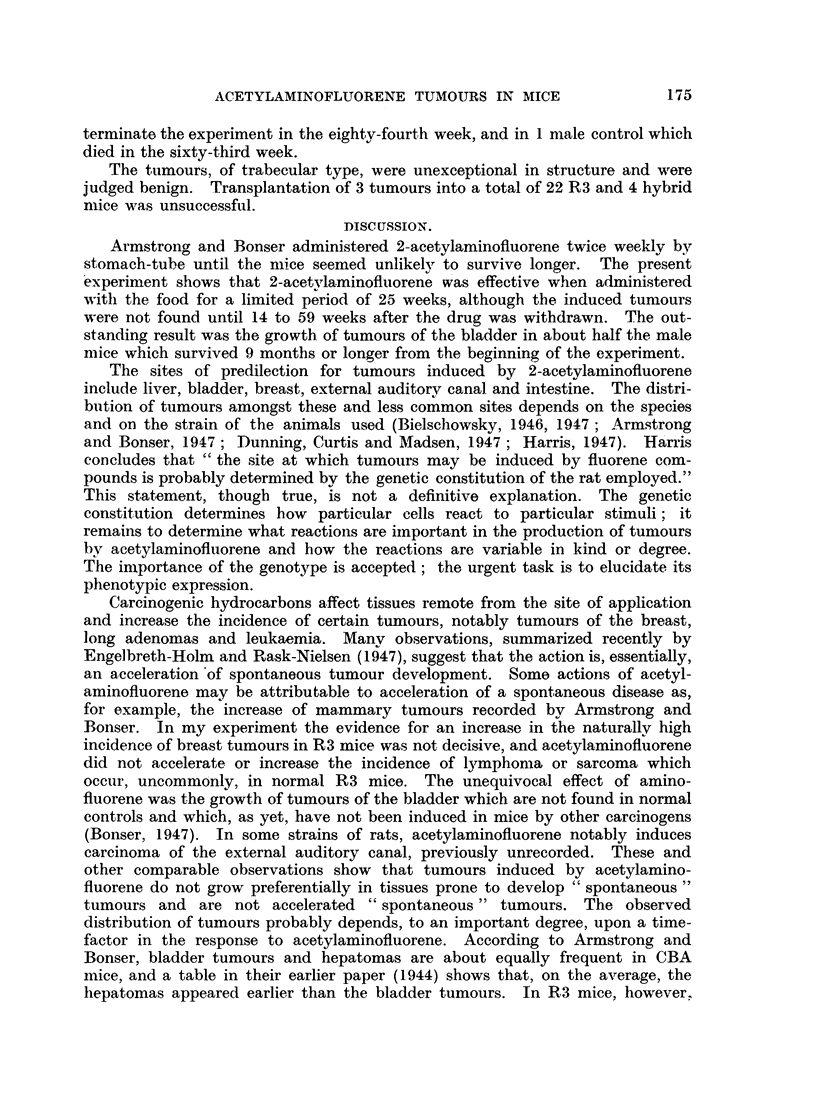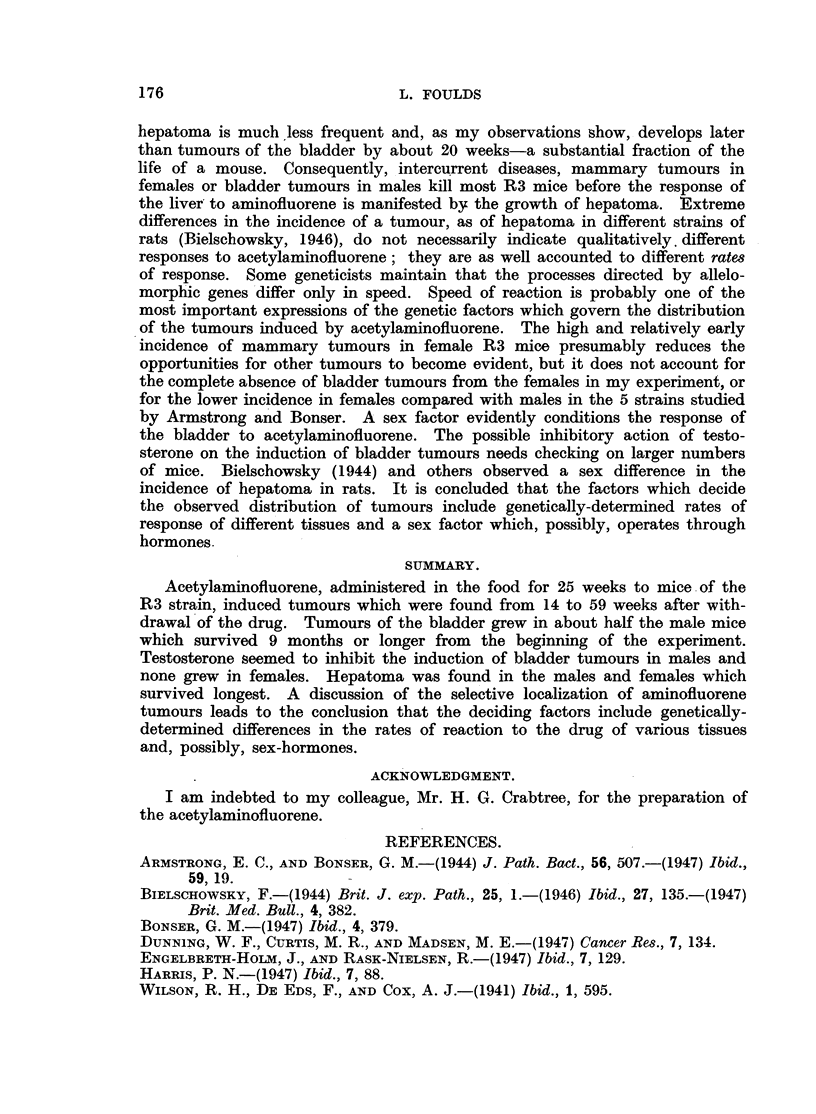# The Induction of Tumours in Mice of the R3 Strain by 2-Acetylaminofluorene

**DOI:** 10.1038/bjc.1947.20

**Published:** 1947-06

**Authors:** L. Foulds


### THE INDUCTION OF TUMOURS IN MICE OF THE R3 STRAIN BY 2-ACETYLAMINOFLUORENE.

L. FOULDS.

*From the Laboratories of the Imperial Cancer Research Fund, Mill Hill, London, N.W. 7.*

Received for publication June 17, 1947.

WILSON, DE EDS AND COX (1941) found that 2-acetylaminofluorene, given by mouth, induced a variety of tumours in rats, and Bielschowsky (1947) summarized the confirmations and extensions of their observations reported up to 1946. Armstrong and Bonser (1944) first demonstrated a similar action in mice, and recently (1947) described the induction of tumours in five inbred strains. The present paper records observations on one of the strains used by Armstrong and Bonser, and, in the main, confirms and amplifies their results.

### METHOD.

Thirty-six male and 36 female mice of the R3 strain received food containing 2-acetylaminofluorene (AAF) and 49 male and 44 female mice from the same stock were kept as controls. Dobrovolskaïa-Zavadskaïa supplied the R3 mice which founded the line maintained in this laboratory by 45 generations of brother-sister matings since 1934. The exact ages of the mice were not known, but were believed to average about 3 months at the beginning of the experiment, at which time the sexes were separated.

The AAF was mixed with food prepared by grinding to powder the Thorley Rat Cubes used in this laboratory as the standard diet for rats and mice, or, during part of the experiment, was mixed with similar food supplied in powder form by the makers of the cubes. The concentration of AAF in the food was 0·03 per cent for 5 weeks and then 0·05 per cent for 20 weeks, the total period of administration extending over 25 weeks from October, 1945, to April, 1946. Thereafter the mice received the standard diet of Rat Cubes for the rest of their lives and the control mice received that diet throughout. All mice had an unrestricted supply of food and water. The experiment was terminated in the eighty-fourth week by killing the mice then surviving, namely 2 males which had eaten AAF and 3 control males.

Several attempts to control the localization of tumours were made on small groups of mice. During the third to sixth weeks of experiment 5 females of the AAF group and 5 control females were mated with vasectomized males and silk threads were tied in the pseudo-pregnant uterine horns to elicit deciduomata. In the twenty-second week of experiment, pellets containing 25 per cent of diethylstilboestrol (B.D.H.) in cholesterol and weighing 5 or 6 mg. were implanted subcutaneously in 9 females of the AAF group and in 10 control females. Disc-shaped tablets containing 5 mg. testosterone propionate (B.D.H.) compressed with 15 mg. calcium lactate were implanted subcutaneously, during the seventeenth and twentieth weeks of experiment, in 9 AAF males and in 10 control males. At various times 10 mammary tumours were excised from 7 AAF females with the primary object, dubiously achieved, of prolonging life to allow time for other tumours to develop.

### RESULTS.

The heaviest mortality attributable with probability to the AAF occurred during the first few weeks of administration, when 11 males and 8 females died. Subsequently the toxic action of AAF was negligible.

No tumours grew in operation scars. The stilboestrol pellets shortened life, especially by causing pyometra. The testosterone tablets were harmless; their possible effect on the induction of bladder tumours is mentioned below. Granulation tissue, fibrosis and abscess were found in uterine horns where silk threads were tied but tumours were not recognized there.

"Spontaneous" tumours were found in 3 male control mice as follows: osteogenic sarcoma of the thigh, with secondary deposits in liver and lungs, in the forty-eighth week; hepatoma in the sixty-third week; and lymphoma in the sixty-eighth week. One female control had lymphoma in the sixtieth week, and many had mammary tumours as described below.

The AAF group provided single examples of (*a*) carcinoma of the small intestine with a secondary deposit in a mesenteric lymph node in the fifty-second week in a male which also had a tumour of the bladder, and (*b*) in the eighty-second week, again in a male with a tumour of the bladder, juxta-renal tumours of lymphoid structure.

The tumours requiring fuller mention were mammary tumours, tumours of the bladder and hepatoma. Table I shows the times of observation of the first tumour of each kind, the number of mice ("effective total") surviving at those times, and the number of tumours. Only tumours visible to the naked eye are included.

TABLE I.

| Type of tumour. | Earliest tumour (weeks of experiment). | Sex. | AAF mice. Effective number of mice. | AAF mice. Number of tumours. | Control mice. Effective number of mice. | Control mice. Number of tumours. |
|---|---|---|---|---|---|---|
| Mammary | 11 | ♂ | 25 | 0 | 45 | 0 |
| | | ♀ | 28 | 17 | 42 | 22 |
| Bladder | 39 | ♂ | 23 | 12 | 44 | 0 |
| | | ♀ | 13 | 0 | 19 | 0 |
| Hepatoma | 60 | ♂ | 9 | 2 | 41 | 1 |
| | | ♀ | 4 | 4 | 6 | 0 |

*Mammary tumours.*

Mammary tumours were found in 60·7 per cent of the effective total of female AAF mice and in 52·4 per cent of the control females. The times of observation ranged from 11 to 61 weeks, with an average of 35·5 weeks in the AAF group, and from 12 to 74 weeks with an average of 40·5 weeks in the controls. Since the exact ages of the mice and their reproductive history before the experiment began are not known, the evidence for an enhancement of mammary tumour development is not convincing. Stilboestrol had no apparent effect on the incidence of tumours, but the number of animals tested was small and soon depleted by deaths from pyometra, and the pellets were implanted at possibly too late a stage of the experiment.

*Tumours of the bladder.*

Tumours of the bladder were found in 12 male mice which received AAF, but not in females which had AAF or in male or female controls. The first of the tumours was found in the thirty-ninth week of experiment and the last in the eighty-second week. The average time was 50·7 weeks, but 8 of the tumours were found from the thirty-ninth to forty-sixth weeks. Only 1 tumour occurred in an effective total of 8 mice carrying testosterone tablets ; 6 of the mice survived from 58 to 64 weeks without tumours. The incidence as a percentage of the effective total was 12·5 for AAF males with testosterone tablets, 73·3 for AAF males without testosterone, and 52·2 for all AAF males.

Most of the tumours were intravesical, pedunculated or sessile papilliferous tumours of transitional- or squamous-cell types, and conformed with the descriptions of Armstrong and Bonser (1944), who distinguished benign and malignant forms but observed no extra-vesical extension. Two tumours of my series extended outside the bladder and disseminated in the abdominal cavity. These two tumours and one which was wholly intra-vesical were transplanted, and consideration of the structural and biological features of the tumours of the bladder is deferred until the transplanted tumours have been further studied.

*Hepatoma.*

The first hepatoma was found in a female of the AAF group killed in the sixtieth week. Three AAF females then survived, and hepatoma was found in each of them during the sixty-second week ; one of them had also a mammary tumour. Hepatoma was present in the 2 AAF males which were killed to terminate the experiment in the eighty-fourth week, and in 1 male control which died in the sixty-third week.

The tumours, of trabecular type, were unexceptional in structure and were judged benign. Transplantation of 3 tumours into a total of 22 R3 and 4 hybrid mice was unsuccessful.

### DISCUSSION.

Armstrong and Bonser administered 2-acetylaminofluorene twice weekly by stomach-tube until the mice seemed unlikely to survive longer. The present experiment shows that 2-acetylaminofluorene was effective when administered with the food for a limited period of 25 weeks, although the induced tumours were not found until 14 to 59 weeks after the drug was withdrawn. The outstanding result was the growth of tumours of the bladder in about half the male mice which survived 9 months or longer from the beginning of the experiment.

The sites of predilection for tumours induced by 2-acetylaminofluorene include liver, bladder, breast, external auditory canal and intestine. The distribution of tumours amongst these and less common sites depends on the species and on the strain of the animals used (Bielschowsky, 1946, 1947 ; Armstrong and Bonser, 1947 ; Dunning, Curtis and Madsen, 1947 ; Harris, 1947). Harris concludes that " the site at which tumours may be induced by fluorene compounds is probably determined by the genetic constitution of the rat employed." This statement, though true, is not a definitive explanation. The genetic constitution determines how particular cells react to particular stimuli ; it remains to determine what reactions are important in the production of tumours by acetylaminofluorene and how the reactions are variable in kind or degree. The importance of the genotype is accepted ; the urgent task is to elucidate its phenotypic expression.

Carcinogenic hydrocarbons affect tissues remote from the site of application and increase the incidence of certain tumours, notably tumours of the breast, long adenomas and leukaemia. Many observations, summarized recently by Engelbreth-Holm and Rask-Nielsen (1947), suggest that the action is, essentially, an acceleration of spontaneous tumour development. Some actions of acetylaminofluorene may be attributable to acceleration of a spontaneous disease as, for example, the increase of mammary tumours recorded by Armstrong and Bonser. In my experiment the evidence for an increase in the naturally high incidence of breast tumours in R3 mice was not decisive, and acetylaminofluorene did not accelerate or increase the incidence of lymphoma or sarcoma which occur, uncommonly, in normal R3 mice. The unequivocal effect of aminofluorene was the growth of tumours of the bladder which are not found in normal controls and which, as yet, have not been induced in mice by other carcinogens (Bonser, 1947). In some strains of rats, acetylaminofluorene notably induces carcinoma of the external auditory canal, previously unrecorded. These and other comparable observations show that tumours induced by acetylaminofluorene do not grow preferentially in tissues prone to develop " spontaneous " tumours and are not accelerated " spontaneous " tumours. The observed distribution of tumours probably depends, to an important degree, upon a time-factor in the response to acetylaminofluorene. According to Armstrong and Bonser, bladder tumours and hepatomas are about equally frequent in CBA mice, and a table in their earlier paper (1944) shows that, on the average, the hepatomas appeared earlier than the bladder tumours. In R3 mice, however, hepatoma is much less frequent and, as my observations show, develops later than tumours of the bladder by about 20 weeks—a substantial fraction of the life of a mouse. Consequently, intercurrent diseases, mammary tumours in females or bladder tumours in males kill most R3 mice before the response of the liver to aminofluorene is manifested by the growth of hepatoma. Extreme differences in the incidence of a tumour, as of hepatoma in different strains of rats (Bielschowsky, 1946), do not necessarily indicate qualitatively different responses to acetylaminofluorene ; they are as well accounted to different *rates* of response. Some geneticists maintain that the processes directed by allelomorphic genes differ only in speed. Speed of reaction is probably one of the most important expressions of the genetic factors which govern the distribution of the tumours induced by acetylaminofluorene. The high and relatively early incidence of mammary tumours in female R3 mice presumably reduces the opportunities for other tumours to become evident, but it does not account for the complete absence of bladder tumours from the females in my experiment, or for the lower incidence in females compared with males in the 5 strains studied by Armstrong and Bonser. A sex factor evidently conditions the response of the bladder to acetylaminofluorene. The possible inhibitory action of testosterone on the induction of bladder tumours needs checking on larger numbers of mice. Bielschowsky (1944) and others observed a sex difference in the incidence of hepatoma in rats. It is concluded that the factors which decide the observed distribution of tumours include genetically-determined rates of response of different tissues and a sex factor which, possibly, operates through hormones.

### SUMMARY.

Acetylaminofluorene, administered in the food for 25 weeks to mice of the R3 strain, induced tumours which were found from 14 to 59 weeks after withdrawal of the drug. Tumours of the bladder grew in about half the male mice which survived 9 months or longer from the beginning of the experiment. Testosterone seemed to inhibit the induction of bladder tumours in males and none grew in females. Hepatoma was found in the males and females which survived longest. A discussion of the selective localization of aminofluorene tumours leads to the conclusion that the deciding factors include genetically-determined differences in the rates of reaction to the drug of various tissues and, possibly, sex-hormones.

### ACKNOWLEDGMENT.

I am indebted to my colleague, Mr. H. G. Crabtree, for the preparation of the acetylaminofluorene.

### REFERENCES.

ARMSTRONG, E. C., AND BONSER, G. M.—(1944) *J. Path. Bact.*, **56**, 507.—(1947) *Ibid.*, **59**, 19.

BIELSCHOWSKY, F.—(1944) *Brit. J. exp. Path.*, **25**, 1.—(1946) *Ibid.*, **27**, 135.—(1947) *Brit. Med. Bull.*, **4**, 382.

BONSER, G. M.—(1947) *Ibid.*, **4**, 379.

DUNNING, W. F., CURTIS, M. R., AND MADSEN, M. E.—(1947) *Cancer Res.*, **7**, 134.

ENGELBRETH-HOLM, J., AND RASK-NIELSEN, R.—(1947) *Ibid.*, **7**, 129.

HARRIS, P. N.—(1947) *Ibid.*, **7**, 88.

WILSON, R. H., DE EDS, F., AND COX, A. J.—(1941) *Ibid.*, **1**, 595.